# Coordinating a Supply Chain with Price and Advertisement Dependent Stochastic Demand

**DOI:** 10.1155/2013/315676

**Published:** 2013-12-18

**Authors:** Liying Li, Yong Wang, Xiaoming Yan

**Affiliations:** ^1^Science College, Chongqing Jiaotong University, Chongqing 400074, China; ^2^School of Economics and Business Administration, Chongqing Key Laboratory of Logistics, Chongqing University, Chongqing 400044, China; ^3^School of Computer, Dongguan University of Technology, Dongguan, Guangdong 523808, China

## Abstract

This paper investigates pricing and ordering as well as advertising coordination issues in a single-manufacturer single-retailer supply chain, where the manufacturer sells a newsvendor-type product through the retailer who faces a stochastic demand depending on both retail price and advertising expenditure. Under the assumption that the market demand has a multiplicative functional form, the Stackelberg and cooperative game models are developed, and the closed form solution to each model is provided as well. Comparisons and insights are presented. We show that a properly designed revenue-cost-sharing contract can achieve supply chain coordination and lead to a Pareto improving win-win situation for channel members. We also discuss the allocation of the extra joint profit according to individual supply chain members' risk preferences and negotiating powers.

## 1. Introduction

### 1.1. Motivation

Advertising is one of the important promotional tools of modern marketing management, by which the manufacturer can influence potential customers and raise brand awareness and the retailer can stimulate the customers' buying behavior [1]. According to the Statistical Abstracts of the United States, the total advertising expenditure in the US had grown from 130 billion dollars in 1990 to about 236 billion dollars in 2000. Even at the individual level of the firm, it is found that in 2002 the largest advertiser (General Motors Corp.) spent more than three billion dollars on advertising ($3,652,000,000 to be exact), while the 100th largest advertiser in the US spent $312,000,000 [2]. Most of the models studying two-tier advertising issue concentrate on the deterministic advertising response function (i.e., the demand function). Little attention has been given to the scenario where the market demand is a stochastic variable depending on advertising expenditure. However, with the rapid improvement of technology, the lifecycles of products nowadays have become shorter and shorter so that more and more products have the attributes of fashion or seasonal goods [3]. These products have common characteristics, such as rapid product substitution, uncertain market demand, and rapid price change.

This paper investigates pricing and ordering as well as advertising expenditure coordination issues for a newsvendor-type product in a single-manufacturer single-retailer supply chain. The manufacturer sells a single-period product through the retailer, who faces a stochastic demand depending on both retail price and advertising expenditure. Before the start of the selling period, the retailer needs to determine the retail price, expenditure on local advertising, and order batch size from the manufacturer, while the manufacturer needs to determine the expenditure on national advertising. Under the assumption that the market demand has a multiplicative functional form, this paper aims to characterize the optimal decisions that each party should adopt and to provide an appropriate incentive scheme to coordinate the whole supply chain.

Coordination among manufacturers (suppliers) and retailers is a very important strategic issue in supply chain management. With supply chain coordination, the upstream supply chain member—the manufacturer (supplier) offers a set of appropriate contract parameters to the retailer such that the retailer's self-profit maximizing objective when making decisions is aligned with the objective of the whole supply chain. Revenue-sharing contract is a widely used effective coordination mechanism proposed by Cachon [4]. Under a revenue-sharing contract, a retailer pays a supplier a wholesale price for each unit purchased, plus a percentage of the revenue the retailer generates. Such contracts have become more prevalent in the videocassette rental industry [5]. However, Cachon [4] demonstrated that revenue-sharing contract fails to coordinate a supply chain with effort-dependent demand. Based on widely-used royalty payments, Kunter [6] designed a cost and revenue-sharing contract to establish efficiency in a manufacturer-retailer channel with deterministic advertising response function. Based on the analysis of the underlying vertical externalities, he showed that channel coordination required cost and revenue-sharing via a revenue-sharing rate and marketing effort participation rates on both manufacturer and retailer level. In this study, we will verify whether a properly designed revenue-cost-sharing contract can coordinate the supply chain with price and advertisement dependent stochastic demand, and has the opportunity to achieve a win-win outcomearisen and under what conditions.

### 1.2. Literature Review

Cooperative (co-op) advertising is a coordinated effort by all members in a distribution channel to increase the customer demand and the overall profits. Some of the early works in this research stream focused on the joint profit maximization problem by determining the optimal brand name investment, local advertising effort, and the participation rate, that is, the percentage of the retailer's local advertising expenditures that the manufacturer agrees to pay (see, e.g., Berger [7]; Somers et al. [8]; Dant and Berger [9]; and Bergen and John [10]). In order to reflect this new market phenomenon, many studies have recently used the game theoretical models to analyze the role of co-op advertising in supply chain coordination. Motivated by observing the power shift from manufacturers to retailers in recent years, represented by the rise of Wal-Mart, Huang and Li [1] and Huang et al. [11] developed and compared two models to reflect different power structure and the corresponding ways of coordinating advertisement spending. The work of Huang et al. [11] was extended by Yue et al. [12], who considered a price-sensitive demand and studied the impact direct price discount from the manufacturer to customers on the channel coordination. Szmerekovsky and Zhang [13] considered pricing and advertising in a two-member supply chain, where customer demand depends on both retail price and advertisement. They obtained both the manufacturer and the retailer's optimal decisions by solving the Stackelberg-manufacturer game. Xie and Neyret [14], Xie and Wei [15], and Seyedesfahani et al. [16] followed a similar approach. They compared the cooperative game optimal results with those of noncooperative. Xie and Neyret [14] and Seyedesfahani et al. [16] investigated four game models, three of which were noncooperative and one was cooperative; whereas Xie and Wei [15] only considered two game models including Stackelberg-manufacturer and cooperative game. Other differences among these papers are to use different demand-price functions.

All the above mentioned models, which studied advertising issue, concentrated on the deterministic advertising response function and studied supply chain coordination problems from cooperative advertising angle. There are some researchers considering the scenario where the market demand is a stochastic variable depending on advertising expenditure. Gerchak and Parlar [17] incorporated the advertising effect (or sales effort) into the newsvendor problem. Under the assumption that the practical market demand is the product of the potential market size and the “diminishing returns on effort” term, they developed a model for simultaneously determining the optimal order quantity and advertising expenditure. Khouja and Robbins [18] extended the single-period problem to a case in which advertising expenditure led to the increase in sales. They dealt with two objectives: maximizing the expected profit and maximizing the probability of achieving a target profit. The above two papers concentrated on finding the optimal policies from the buyer's perspective. Recently, Chen [19] investigated the combined effects of the cooperative advertising mechanism, the return policy, and the channel coordination for a single-period commodity in a two-level supply chain. In his study, the market demand was independent of retail price and a profit-sharing mechanism based on achieving a win-win relationship of the channel members was proposed to coordinate the supply chain. This is different from the model we consider in this paper, where the pricing, ordering, and advertising decisions in the supply chain coordination are simultaneously considered. In our study, the market demand is stochastic and influenced by both retail price and advertising efforts by channel members. In addition, a revenue-cost-sharing contract is used to coordinate the whole supply chain.

The remainder of the paper is organized as follows.  Section 2 introduces notations and assumptions needed to develop the model. In Section 3, the optimal policies of cooperative game model are provided and the impact of demand uncertainty on the optimal policies is discussed. In Section 4, the optimal policies of Stackelberg game are presented, and the optimal strategies and system's expected profits in the two game equilibriums are compared.  Section 5 presents a revenue-cost-sharing contract that can realize coordination of the channel and discusses the allocation of the extra joint profit between channel members.  Section 6 presents the numerical examples to illustrate the models. Finally, conclusions are given in Section 7.

## 2. Notations and Assumptions

We study a single-period supply chain consisting of a manufacturer and a retailer where the manufacturer sells a single-period product through the retailer. The retailer faces a stochastic demand depending on both retail price and advertising efforts by the manufacturer and the retailer.

The following notations and assumptions are used.

### 2.1. Notations

 
*p*: The retailer's unit retail price (decision variable).
 
*n*: The national advertising expenditure of the manufacturer (decision variable).
 
*e*: The local advertising expenditure of the retailer (decision variable).
 
*q*: The retailer's order quantity (decision variable).
 
*w*: The unit wholesale price of the manufacturer.
 
*c*_*r*_: The unit retail cost of the retailer.
 
*c*_*m*_: The unit production cost of the manufacturer.
 
*c*: The unit total channel cost, *c* = *c*_*r*_ + *c*_*m*_.
 
*D*(*p*, *n*, *e*): The expected value of demand for the product.
 
*d*(*p*), *g*(*n*, *e*): The deterministic functions which reflect the influences of the retail price and advertising expenditure on the expected demand, respectively.
 
*ε*: The positive stochastic variable with mean equal to one, and CDF *F*(*x*) and PDF *f*(*x*).
 
*θ*: The retailer's share of the national advertising expenditure, 0 < *θ* < 1.
 
*φ*: The manufacturer's share of the local advertising expenditure, 0 < *φ* < 1.
 
*λ*: The manufacturer's share of revenue generated from each unit, 0 < *λ* < 1.

### 2.2. Assumptions

(1) The practical demand for the product, denoted by *x*, is defined as the product of *D*(*p*, *n*, *e*) and *ε*, where *ε* is a positive stochastic variable with mean equal to one, that is, *x* = *D*(*p*, *n*, *e*) · *ε*. Assume that the probability distribution has support on [*A*, *B*] with *B* > *A* ≥ 0. Let *h*(*x*) ≡ *xf*(*x*)/(1 − *F*(*x*)) denote the generalized failure rate of the demand distribution. We assume that *dh*(*x*)/*dx* > 0; that is, the stochastic variable *ε* has an increasing generalized failure rate. This property is known to be satisfied by common distributions like normal, uniform, the gamma, and Weibull families [20]. Similar to many models studying advertising issue, the expected demand *D*(*p*, *n*, *e*) is modeled in a multiplicative form (see, e.g., Yue et al. [12]; Szmerekovsky and Zhang [13]; Xie and Neyret [14]; Xie and Wei [15]; and Seyedesfahani et al. [16]); that is,

(1) $$\begin{array}{c} D\left(p,n,e\right)=d\left(p\right)g\left(n,e\right). \end{array}$$

We let *d*(*p*) take the form of

(2) $$\begin{array}{c} d\left(p\right)=ap^{-b}, \end{array}$$

where *a* is a measurement of market scale (*a* > 0) and *b* represents the price elasticity of market demand. We focus on price-elastic products by assuming *b* > 1, which has been used by many researchers, such as Wang et al. [21] and Li et al. [22]. The advertising effect *g*(*n*, *e*) is taken in the similar form as mentioned by Xie and Wei [15] and Seyedesfahani et al. [16]. Consider

(3) $$\begin{array}{c} g\left(n,e\right)=k_{1}\sqrt{e}+k_{2}\sqrt{n}, \end{array}$$

where *k*_1_ and *k*_2_ are positive constants that, respectively, reflect the efficacy of each type of advertising in generating sales. It can be found from (3) that *g*(*n*, *e*) is an increasing concave function of both *n* and *e*.

(2) For simplicity, we assume that unsold product at the end of the season bears no salvage value or disposal cost. In addition, in the case of shortages, unsatisfied demand carries no additional penalty.

(3) We assume that all the information (e.g., market demand and cost structure) is common knowledge to both parties.

## 3. Cooperative Game Model 

In this section, we focus on a cooperative game structure. That is to say, both the manufacturer and the retailer agree to make decisions to maximize the whole channel's profit. These decisions include the optimal national and local advertising expenditure, the optimal order quantity, and the optimal retail price; and they have to be made before the start of the selling period. Let Π_*T*_(*q*, *p*, *n*, *e*) denote the expected profit of the integrated system; then we have

(4) ΠT(q,p,n,e)=pE[min⁡(D(p,n,e)·ε,q)]−cq−n−e=pE[min⁡(ap−bg(n,e)ε,q)]−cq−n−e,

where *g*(*n*, *e*) is defined in (3).

Following Petruzzi and Dada [23], we define a “stocking factor”: *z* = *q*/[*ap* ^−*b*^ *g*(*n*, *e*)], based on which, the decision variable (*q*, *p*, *n*, *e*) can be transformed to (*q*, *z*, *n*, *e*). By substituting *p* = [*azg*(*n*, *e*)/*q*]^1/*b*^ into (4), the expected profit of the integrated system can be written as

(5) ΠT(q,z,n,e)=(azg(n,e)q)1/bqE[min⁡(εz,1)] −cq−n−e=[azg(n,e)]1/bq1−1/b ×[1−Γ(z)]−cq−n−e,

where

(6) $$\begin{array}{c} \Gamma \left(z\right)=\overset{z}{\underset{A}{\int }}\left(1-\frac{x}{z}\right)f\left(x\right)dx. \end{array}$$

**Theorem 1**

The cooperative game has the following optimal solution. (1) The optimal stocking factor, *z*_T_*, is determined by

(7) $$\begin{array}{c} F\left(z\right)=\frac{z+\left(b-1\right)\Lambda \left(z\right)}{bz}, \end{array}$$

where

(8) $$\begin{array}{c} \Lambda \left(z\right)=\overset{z}{\underset{A}{\int }}\left(z-x\right)f\left(x\right)dx. \end{array}$$

If *dh*(*x*)/*dx* > 0, then the optimal stocking factor *z*_T_* is unique.

(2) The unique optimal local advertising expenditure, *e*_T_*, is given by

(9) $$\begin{array}{c} e_{T}^{*}={\left\{\frac{ak_{1}z_{T}^{*}{\left[1-F\left(z_{T}^{*}\right)\right]}^{b}}{2\left(b-1\right)c^{b-1}}\right\}}^{2}. \end{array}$$

(3) The unique optimal national advertising expenditure, *n*_T_*, is given by

(10) $$\begin{array}{c} n_{T}^{*}={\left\{\frac{ak_{2}z_{T}^{*}{\left[1-F\left(z_{T}^{*}\right)\right]}^{b}}{2\left(b-1\right)c^{b-1}}\right\}}^{2}. \end{array}$$

(4) The unique optimal order quantity, *q*_T_*, is given by

(11) $$\begin{array}{c} q_{T}^{*}=\frac{\left(k_{1}^{2}+k_{2}^{2}\right){\left[az_{T}^{*}{\left(1-F\left(z_{T}^{*}\right)\right)}^{b}\right]}^{2}}{2\left(b-1\right)c^{2b-1}}. \end{array}$$

**Proof**

See Appendix A.

From Theorem 1, we find that in the cooperative game, the optimal stocking factor *z*_T_* is only determined by the price elasticity *b* and the distribution of stochastic variable *ε* and is not related to other parameters. This greatly simplifies the process of our model. The optimal national and local advertising expenditures are dependent on the corresponding efficacy coefficient *k*_1_ and *k*_2_. The optimal order quantity is simultaneously dependent on the efficacy coefficient of each type of advertising. The ways by which *e*_T_*, *n*_T_*, and *q*_T_* depend on *b* are more complex and will be characterized through the numerical studies. Additionally, we find that the ratio of the optimal national and local advertising expenditures is *k*_2_^2^/*k*_1_^2^.

Once (*q*_T_*, *n*_T_*, *e*_T_*) is determined, it is not difficult to find the optimal retail price. Substituting (9)–(11) into *p* = [*azg*(*n*, *e*)/*q*]^1/*b*^, the optimal retail price is expressed by

(12) $$\begin{array}{c} p_{T}^{*}=\frac{c}{1-F\left(z_{T}^{*}\right)}. \end{array}$$

From (12), it is easy to find that in the cooperative game, the optimal retail price is independent of the price elasticity and efficacy coefficient of each type of advertising.

Substituting (9)–(11) into (5), we obtain the optimal expected profit of the integrated system, Π_T_*, as follows:

(13) $$\begin{array}{c} \Pi_{T}^{*}=\frac{cq_{T}^{*}}{b-1}-n_{T}^{*}-e_{T}^{*}. \end{array}$$

### 3.1. The Impact of Demand Uncertainty on the Optimal Policies

If demand is only influenced by retail price and advertising expenditure and has no uncertainty, then the demand faced by the retailer can be expressed as *D*(*p*, *n*, *e*). Under such a deterministic case, the order quantity of the retailer is equal to the demand of the market; that is, *q* = *D*(*p*, *n*, *e*). Hence, the total profit of the integrated system, denoted as Π_*T*_^det⁡^, can be given by

(14) ΠTdet⁡=(p−c)D(p,n,e)−n−e=ap−b(p−c)(k1e+k2n)−n−e.

Maximizing Π_*T*_^det⁡^ in (14), one can easily derive the optimal policies under the deterministic setting as follows:

(15) $$\begin{array}{c} e_{T}^{\det}={\left\{\frac{ak_{1}{\left(b-1\right)}^{b-1}}{2b^{b}c^{b-1}}\right\}}^{2}, \end{array}$$

(16) $$\begin{array}{c} n_{T}^{\det}={\left\{\frac{ak_{2}{\left(b-1\right)}^{b-1}}{2b^{b}c^{b-1}}\right\}}^{2}, \end{array}$$

(17) $$\begin{array}{c} p_{T}^{\det}=\frac{bc}{b-1}. \end{array}$$

By comparing the optimal policies obtained under the deterministic and stochastic settings, we obtain the following results.

**Theorem 2**

*e*_*T*_^det⁡^ > *e*_T_*, *n*_*T*_^*det*^ > *n*_T_*, *p*_*T*_^*det*^ < *p*_T_*, and *D*(*p*_T_*, *n*_T_*, *e*_T_*) < *D*(*p*_*T*_^*det*^, *n*_*T*_^*det*^, *e*_*T*_^*det*^).

**Proof**

See Appendix B.

Theorem 2 indicates that, comparing with certainty environment, the uncertainty of the demand will lead to the decrease of advertising expenditure and the enhancement of retail price, which ultimately reduce the expected sales of the product.

## 4. Stackelberg Game Model

In this section, the relationship between the manufacturer and the retailer is modeled as a sequential noncooperative game, where the manufacturer is the leader and the retailer is the follower. In this setting, the retailer would determine simultaneously the local advertising expenditure, retail price, and order quantity to maximize her own profit, and the manufacturer would determine the national advertising expenditure and wholesale price based on the retailer's decision.

As in Section 3, we define the stocking factor of inventory as *z* = *q*/[*ap* ^−*b*^ *g*(*n*, *e*)]. Then, choosing (*q*, *p*, *n*, *e*) is equivalent to choosing (*q*, *z*, *n*, *e*). Denoting the retailer's expected profit function by Π_*r*_(*q*, *p*, *e*), we have

(18) Πr(q,p,e)=pE[min⁡(D(p,n,e)·ε,q)]−(cr+w)q−e=[azg(n,e)]1/bq1−1/b[1−Γ(z)]−(cr+w)q−e,

where *g*(*n*, *e*) and Γ(*z*) are defined in (3) and (6), respectively.

Similar to the induction of Theorem 1, we can show that the unique optimal response for the retailer is as follows: (1) the optimal stocking factor *z*_d_* is equal to that of the cooperative game; that is, *z*_d_* = *z*_T_*; (2) the local advertising, ordering, and pricing strategies are given by

(19) $$\begin{array}{c} e_{d}\left(w\right)={\left\{\frac{ak_{1}z_{d}^{*}{\left[1-F(z_{d}^{*})\right]}^{b}}{2(b-1){\left(w+c_{r}\right)}^{b-1}}\right\}}^{2}, \end{array}$$

(20) $$\begin{array}{c} q_{d}\left(w\right)=az_{d}^{*}\left(k_{1}\sqrt{e_{d}\left(w\right)}+k_{2}\sqrt{n}\right){\left[\frac{\left(1-F\left(z_{d}^{*}\right)\right)}{\left(w+c_{r}\right)}\right]}^{b}, \end{array}$$

(21) $$\begin{array}{c} p_{d}\left(w\right)=\frac{w+c_{r}}{1-F\left(z_{d}^{*}\right)}. \end{array}$$

From (19)–(21), it can be found that the retailer's retail price and local advertising expenditure are not affected by the manufacturer's national advertising expenditure *n*, but the order quantity is increasing in *n*.

The manufacturer's expected profit function, denoted by Π_*m*_(*w*, *n*), can be given by

(22) $$\begin{array}{c} \Pi_{m}\left(w,n\right)=\left(w-c_{m}\right)q-n. \end{array}$$

Substituting (19) and (20) into (22), we obtain

(23) Πm(w,n)=M(w−cm)(w+cr)−b ×[k1N(w+cr)1−b+k2n]−n,

where

(24) $$\begin{array}{c} M=az_{d}^{*}{\left[1-F\left(z_{d}^{*}\right)\right]}^{b}, \end{array}$$

(25) $$\begin{array}{c} N={\left\{\frac{ak_{1}z_{d}^{*}{\left[1-F(z_{d}^{*})\right]}^{b}}{2(b-1)}\right\}}^{2}. \end{array}$$

Solving the manufacturer's decision problem (23) and considering (19)–(21), we can obtain the Stackelberg game equilibrium.

**Theorem 3**

The Stackelberg game has the following unique equilibrium. (1) The optimal wholesale price, w*, is given by

(26) $$\begin{array}{c} w^{*}=\frac{c_{r}+yc_{m}}{y-1}. \end{array}$$

(2) The optimal national advertising expenditure, *n*_d_*, is given by

(27) $$\begin{array}{c} n_{d}^{*}={\left\{\frac{ak_{2}z_{d}^{*}{\left(y-1\right)}^{b-1}{\left[1-F(z_{d}^{*})\right]}^{b}}{2y^{b}c^{b-1}}\right\}}^{2}. \end{array}$$

(3) The optimal local advertising expenditure, *e*_d_*, is given by

(28) $$\begin{array}{c} e_{d}^{*}={\left\{\frac{ak_{1}z_{d}^{*}{\left(y-1\right)}^{b-1}{\left[1-F(z_{d}^{*})\right]}^{b}}{2(b-1){\left(yc\right)}^{b-1}}\right\}}^{2}. \end{array}$$

(4) The optimal order quantity, *q*_d_*, is given by

(29) $$\begin{array}{c} q_{d}^{*}=\frac{\left[yk_{1}^{2}+\left(b-1\right)k_{2}^{2}\right]{\left[az_{d}^{*}{\left(1-F(z_{T}^{*})\right)}^{b}\right]}^{2}{\left(y-1\right)}^{2b-1}}{2\left(b-1\right)c^{2b-1}y^{2b}}. \end{array}$$

(5) The optimal retail price, *p*_d_*, is given by

(30) $$\begin{array}{c} p_{d}^{*}=\frac{yc}{\left(y-1\right)\left[1-F\left(z_{d}^{*}\right)\right]}, \end{array}$$

where

(31) y=[(1−b)k2+2b−1 +k4(b−1)2+2k2(b−1)+(2b−1)2]×2−1,

and *k* = *k*_2_/*k*_1_, which is called the advertising ratio in this paper, reflecting the relative effectiveness of national versus local advertising in generating sales.

**Proof**

See Appendix C.

Substituting (26)–(29) into (18) and (22), respectively, we can show that in the Stackelberg game, the retailer's and manufacturer's profits, denoted by Π_r_* and Π_m_*, are given by

(32) $$\begin{array}{c} \Pi_{r}^{*}=\frac{ycq_{d}^{*}}{\left[\left(b-1\right)\left(y-1\right)\right]}-e_{d}^{*}, \end{array}$$

(33) $$\begin{array}{c} \Pi_{m}^{*}=\frac{cq_{d}^{*}}{y-1}-n_{d}^{*}. \end{array}$$

To compare the optimal policies obtained in the Stackelberg and cooperative game settings, we first introduce the following lemma.

**Lemma 4**

*y* > *b*.

**Proof**

See Appendix D.

By comparing the optimal policies obtained in the Stackelberg and cooperative game settings, we derive the following results.

**Corollary 5**

*e*_d_* < *e*_T_*,  *n*_d_* < *n*_T_*, *p*_d_* > *p*_T_*, and *q*_d_* < *q*_T_*.

**Proof**

See Appendix E.

Corollary 5 shows that the retail price in the Stackelberg game is always higher than that in the cooperative game; the order quantity and national and local advertising expenditures are always lower. As a consequence, the channel profit in the Stackelberg game is always lower than that in the cooperative game.

For the rest of this section we provide some properties that characterize the optimal decisions in the two games. From (9), (10), (27), (28), and Lemma 4, we can easily derive the following Property 1.

**Property 1**

*n*_d_*/*e*_d_* < *n*_T_*/*e*_T_*.

Property 1 shows that the ratio of the optimal national and local advertising expenditures in the Stackelberg game is lower than that in the cooperative game.

From (26) and (30), we can obtain the following Property 2.

**Property 2**

dw*/*dk* > 0 and *dp*_d_*/*dk* > 0.

**Proof**

See Appendix F.

Property 2 shows that in the Stackelberg game, both the optimal wholesale price and the retail price are increasing in the advertising ratio *k*.

From (27)–(29), we can easily derive the following Property 3.

**Property 3**

*dn*_d_*/*dk*_2_ > 0, *de*_d_*/*dk*_1_ > 0,  ∂*q*_d_*/∂*k*_1_ > 0, and ∂*q*_d_*/∂*k*_2_ > 0.

The proofs of Properties 1 and 3 can be easily obtained and hence are omitted for simplicity.

Property 3 shows that in the Stackelberg game, the optimal national advertising expenditure increases as the efficacy of national advertising increases. The same monotonic pattern can apply for the optimal local advertising expenditure. Additionally, the optimal order quantity increases as the efficacy of each type of advertising increases. Note that Property 3 also holds for the optimal ordering and advertising policies in the cooperative game.

## 5. Supply Chain Coordination

According to Corollary 5, the channel profit in the Stackelberg game is always lower than that in the cooperative game. This phenomenon is well known as “double marginalization” [4]. To encourage the retailer to order more and spend more money on advertising, the manufacturer will offer appropriate contract parameters to the retailer so that the supply chain is coordinated. With supply chain coordination, the retailer's self-profit maximizing objective when making decisions is aligned with the objective of the whole supply chain.

In the following, we first discuss how a revenue-cost-sharing contract coordinates the supply chain presented in our model and then utilize the Eliashberg's model to determine the allocation of the cooperative profit.

### 5.1. Revenue-Cost Sharing Contract

We assume that the manufacturer and the retailer consider a revenue-cost-sharing contract (*λ*, *w*, *φ*, *θ*), under which the manufacturer (retailer) agrees to bear a fraction *φ*(*θ*) of advertising cost of the retailer (manufacturer), and the manufacturer would share a fraction *λ* of the retailer's sales revenue.

Under the revenue-cost-sharing contract, the expected profit functions of the manufacturer and retailer, denoted by Π_*r*_^RC^(*q*, *p*, *n*, *e*) and Π_*m*_^RC^(*q*, *p*, *n*, *e*), respectively, can be expressed as

(34) ΠrRC(q,p,n,e)=(1−λ)pE[min⁡(D(p,n,e)·ε,q)] −(w+cr)q−θn−(1−φ)e,

(35) ΠmRC(q,p,n,e)=λpE[min⁡(D(p,n,e)·ε,q)] +(w−cm)q−(1−θ)n−φe.

**Theorem 6**

A revenue-cost-sharing contract can coordinate the supply chain facing a stochastic demand depending on both retail price and advertising expenditures, if the following conditions hold:

(36) $$\begin{array}{c} w=c_{m}-\lambda c,\\ 1-\theta =\lambda =\varphi . \end{array}$$

**Proof**

Substituting (36) into (34) and (35) and simplifying, we have

(37) $$\begin{array}{c} \Pi_{r}^{\text{RC}}\left(q,p,n,e\right)=\left(1-\lambda \right)\Pi_{T}\left(q,p,n,e\right),\\ \Pi_{m}^{\text{RC}}\left(q,p,n,e\right)=\lambda \Pi_{T}\left(q,p,n,e\right). \end{array}$$

From (37), it follows that, given *n*_T_*, (*q*_T_*, *p*_T_*, *e*_T_*) maximizes the retailer's profit when *λ* < 1, and given (*q*_T_*, *p*_T_*, *e*_T_*), *n*_T_* maximizes the manufacturer's profit when *λ* > 0. Hence, the proof of Theorem 6 is completed.

From (36) in Theorem 6, one can easily observe the following.

- Under the revenue-cost-sharing contract, the manufacturer's wholesale price is lower than its corresponding unit marginal cost *c*_*m*_, which means that the manufacturer earns profit from the retailer's sales revenue rather than his own sales income.
- Under the revenue-cost-sharing contract, the manufacturer's share of his own advertising cost equals the share of the retailer's advertising cost and the share of the retailer's revenue.

From (37), one can find that the manufacturer's expected profit is increasing in *λ* and the retailer's expected profit is decreasing in *λ*. The manufacturer earns all the joint system profit when *λ* = 1, and the retailer earns all the joint system profit when *λ* = 0. Hence, revenue-cost-sharing contract can arbitrarily allocate the joint system profit between two parties.

**Theorem 7**

Under a revenue-cost-sharing contract with parameters satisfying (36), the manufacturer and the retailer get a profit larger than that in the Stackelberg game if the following conditions hold:

(38) $$\begin{array}{c} \lambda_{\min}<\lambda <\lambda_{\max}, \end{array}$$

where

(39) $$\begin{array}{c} \lambda_{\min}=\frac{\Pi_{m}^{*}}{\Pi_{T}^{*}},\;\;\lambda_{\max}=\frac{\Pi_{T}^{*}-\Pi_{r}^{*}}{\Pi_{T}^{*}}. \end{array}$$

**Proof**

From (37)–(39), we get Π_*r*_^RC^ = (1 − *λ*)Π_T_* > Π_T_*[1 − (Π_T_* − Π_r_*)/Π_T_*] = Π_r_*, and Π_*m*_^RC^ = *λ*Π_T_* > Π_T_*(Π_m_*/Π_T_*) = Π_m_*. Hence, the proof of Theorem 7 is completed.

Theorem 7 shows that a revenue-cost-sharing contract with parameters satisfying (36) and (38) can not only achieve supply chain coordination, but can also lead to a Pareto improving win-win situation for channel members.

### 5.2. Bargaining Problem

In this subsection, we discuss the selection of the optimal revenue-cost-sharing contract which realizes the allocation of the extra joint profit.

Economic literature on bargaining theory is based on the seminal papers by Nash [24]. It is worth pointing out that Nash's bargaining model does not take individual members' negotiating powers into account while predicting the implementation outcome of a contract, which is a severe deficiency of the model, because the selection of a contract clearly depends on channel members' negotiating powers. In order to overcome this deficiency, an alternative way is to apply the approach introduced by Eliashberg [25]. Eliashberg's model is used to predict a revenue-cost-sharing contract that maximizes the system utility function reflecting the joint preferences of channel members. By Eliashberg's model, we can incorporate channel members' negotiating powers into the ultimate implementation outcome, with the use of aggregation weights that measure the relative negotiating powers of the supply chain members.

For ease of exposition, we denote

(40) $$\begin{array}{c} \Delta \Pi_{r}=\Pi_{r}^{\text{RC}}\left(\lambda \right)-\Pi_{r}^{*}=\left(1-\lambda \right)\Pi_{T}^{*}-\Pi_{r}^{*},\\ \Delta \Pi_{m}=\Pi_{m}^{\text{RC}}\left(\lambda \right)-\Pi_{m}^{*}=\lambda \Pi_{T}^{*}-\Pi_{m}^{*}, \end{array}$$

where ΔΠ_*r*_ and ΔΠ_*m*_ correspond to the additional profits split by the retailer and the manufacturer from ΔΠ, which are the system's increased profits from coordination with the revenue-costs-sharing contract associated with parameters satisfying (36) and (38). Clearly, ΔΠ_*r*_ + ΔΠ_*m*_ = ΔΠ for all *λ*.

Consider the following exponential utility functions for the retailer and the manufacturer:

(41) $$\begin{array}{c} U_{r}\left(\Delta \Pi_{r}\right)=-\exp\left(-\phi_{r}\Delta \Pi_{r}\right),\\ U_{m}\left(\Delta \Pi_{m}\right)=-\exp\left(-\phi_{m}\Delta \Pi_{m}\right), \end{array}$$

where *ϕ*_*r*_, *ϕ*_*m*_ > 0. By the Pratt-Arrow risk aversion functions [26], it is easy to know that a larger *ϕ*_*r*_ or *ϕ*_*m*_ indicates a more risk-averse member. We suppose the retailer's relative bargaining power is measured by *λ*_*r*_ and the manufacturer's by *λ*_*m*_. Without loss of generality, we assume *λ*_*r*_ + *λ*_*m*_ = 1. To obtain the Eliashberg's solution, the following programming problem needs to be solved:

(42) max⁡λ Us(ΔΠm,ΔΠr)=λmUm(ΔΠm)+λrUr(ΔΠr)=−λmexp⁡⁡(−ϕmΔΠm)−λrexp⁡(−ϕrΔΠr),s.t.λ∈[λmin⁡,λmax⁡],

where *U*_*s*_(ΔΠ_*m*_, ΔΠ_*r*_) denotes the system's utility function.

Solving the programming problem (42), we obtain the optima revenue-cost-sharing contract parameter, λ*, as follows.

(i) If *λ*_*m*_/*λ*_*r*_ ≤ *ϕ*_*r*_/[*ϕ*_*m*_exp⁡(*ϕ*_*r*_ΔΠ)], then λ* = *λ*_min⁡_ and the retailer will obtain all the extra profit ΔΠ;
(ii) If *λ*_*m*_/*λ*_*r*_ ≥ *ϕ*_*r*_exp⁡(*ϕ*_*m*_ΔΠ)/*ϕ*_*m*_, then λ* = *λ*_max⁡_ and the manufacturer will gain all the extra profit ΔΠ;
(iii) If *λ*_*m*_/*λ*_*r*_ ∈ [*ϕ*_*r*_/(*ϕ*_*m*_exp⁡(*ϕ*_*r*_ΔΠ)), *ϕ*_*r*_exp⁡(*ϕ*_*m*_ΔΠ)/*ϕ*_*m*_], then
  (43) λ∗=ϕrϕr+ϕmλmax⁡+ϕmϕr+ϕmλmin⁡+1(ϕr+ϕm)ΠT∗ln⁡⁡λmϕmλrϕr,

and the allocation of the extra profit ΔΠ is as follows:

(44) $$\begin{array}{c} \Delta \Pi_{m}=\frac{\phi_{r}}{\phi_{r}+\phi_{m}}\Delta \Pi +\frac{1}{\phi_{r}+\phi_{m}}\ln\frac{\lambda_{m}\phi_{m}}{\lambda_{r}\phi_{r}},\\ \Delta \Pi_{r}=\frac{\phi_{m}}{\phi_{r}+\phi_{m}}\Delta \Pi -\frac{1}{\phi_{r}+\phi_{m}}\ln\frac{\lambda_{m}\phi_{m}}{\lambda_{r}\phi_{r}}. \end{array}$$

From (44), we can find that for such a coordinated supply chain, the retailer will obtain a share *ϕ*_*m*_/(*ϕ*_*r*_ + *ϕ*_*m*_) from the extra profit ΔΠ and the manufacturer will gain a share *ϕ*_*r*_/(*ϕ*_*r*_ + *ϕ*_*m*_). A compensation fee between the retailer and the manufacturer is [1/(*ϕ*_*r*_ + *ϕ*_*m*_)]ln⁡(*λ*_*m*_ *ϕ*_*m*_/*λ*_*r*_ *ϕ*_*r*_), which represents a fee paid by the retailer to the manufacturer if *λ*_*m*_/*λ*_*r*_ ≥ *ϕ*_*r*_/*ϕ*_*m*_; otherwise, from the manufacturer to the retailer. From (44), we also see that the proportions shared by the retailer and the manufacturer only depend on their risk aversion measurements *ϕ*_*r*_ and *ϕ*_*m*_ and are unrelated to their relative power measurements *λ*_*r*_ and *λ*_*m*_. The more risk averse a member is, the less of the share it will obtain from the extra profit ΔΠ. Given *ϕ*_*r*_ and *ϕ*_*m*_, an increase in *λ*_*m*_ or a decrease in *λ*_*r*_ means an increasing compensation fee from the retailer to the manufacturer if *λ*_*m*_/*λ*_*r*_ ≥ *ϕ*_*r*_/*ϕ*_*m*_. In other words, with an increase in the relative power of the manufacturer with respect to the retailer, it will receive a higher compensation fee from the retailer. When the manufacturer's relative power is high enough with respect to the retailer (e.g., *λ*_*m*_/*λ*_*r*_ ≥ *ϕ*_*r*_exp⁡(*ϕ*_*m*_ΔΠ)/*ϕ*_*m*_), the result of coordination will be that the manufacturer captures all the extra profit, whereas the retailer receives nothing from coordination. A similar analysis is applicable to the case where *λ*_*m*_/*λ*_*r*_ < *ϕ*_*r*_/*ϕ*_*m*_. Besides, it is worth noting that when the retailer and the manufacturer are equally risk-averse, that is, *ϕ*_*r*_ = *ϕ*_*m*_, they will split the extra profit in equal proportions, and their relative power measurements *λ*_*m*_ and *λ*_*r*_ will be the only factors that decide whether a member receives a positive or negative compensation fee from the other one.

## 6. Numerical Examples

In order to illustrate the model and gain more insights, we implement a numerical study. First, we assume that stochastic variable *ε* with mean equal to one follows the uniform distribution in [*A*, *B*], where *A* = 0 and *B* = 2. For the rest of the parameters of the model, we extract stochastically a value out of its given interval, which is shown in Table 1. We then compute the equilibrium solution of the problem in the two game settings based on this group of extracted values of all parameters. We extract stochastically more than 100 groups of values of the parameters in total in the experiment. All these numerical experiments support the similar conclusion. For brevity, we pick arbitrarily six from all groups, in which the values of parameters are listed in Table 2, to illustrate our observations intuitively.  Table 3 shows their corresponding equilibrium solutions, where Π_*S*_ denotes the whole system's expected profit. From Table 3, one easily observes the similar insights with those observed from Corollary 5. That is, coordination leads to lower retail price, larger advertising expenditure, and larger order quantity. In addition, we define the percentage, Δ, by which the system's expected profit in the cooperative game increases over the system's expected profit in the Stackelberg game as

(45) $$\begin{array}{c} \Delta =\frac{\Pi_{T}^{*}-\left(\Pi_{m}^{*}+\Pi_{r}^{*}\right)}{\Pi_{m}^{*}+\Pi_{r}^{*}}. \end{array}$$

From Table 3, one can easily find that the maximum of Δ is around Δ = 129.0%, suggesting that the value of coordination can be significant.

In what follows, we investigate the influence of changes of parameters *k*_1_, *k*_2_, and *b* on the equilibrium solutions for channel members in the two game settings. The results are shown in Tables 4 and 5. The values of other parameters in Tables 4 and 5 are kept the same as in Group 2.

From Table 4, it can be noted that the regularity of impact of changes of *k*_1_, *k*_2_, and *k* = *k*_2_/*k*_1_ on the optimal policies in two game settings is identical to that presented in Properties 1, 2, and 3, which further verifies Properties 1, 2, and 3 numerically. In addition, from Table 4 we can find that the percentage increase of the system's expected profit decreases as *k* decreases, which shows that the value of coordination decreases as the ratio of the efficacy coefficient of national and local advertising decreases.

From Table 5, it can be seen that in the two game settings, the optimal retail price, order quantity, and national and local advertising expenditures notably decrease as the price elasticity of market demand increases. In addition, the profits of the manufacturer, retailer, and supply chain system notably decrease as the price elasticity increases, but the price elasticity has little impact on the percentage increase of the system's profit.

Table 6 shows that given *λ*_*r*_ and *λ*_*m*_, the more risk averse a member is, the less he obtains from the extra profit. Additionally, given *ϕ*_*r*_ and *ϕ*_*m*_, the retailer (manufacturer) receives more extra profit when his relative bargaining power with respect to the manufacturer (retailer) increases. When both the risk aversion measurements and the relative power measurements of the retailer and the manufacturer are equal, they will split the extra profit equally.

## 7. Conclusions

This paper investigates the coordination problem of pricing, ordering, and advertising for a newsvendor-type product in a supply chain consisting of a manufacturer and a retailer. The consumer demand is influenced by both retail price and advertising expenditures. For a specific demand function with multiplicative functional form, we develop the decision models of the cooperative and Stackelberg games, respectively, and provide the closed form solution to each model. By comparing the optimal policies in the two games, we find that the retail price in the Stackelberg game is always higher than that in the cooperative game, whereas the order quantity and advertising expenditure are always lower. As a consequence, the higher amount of profit for the whole system is achieved in the case of cooperation. We also find that the ratio of the optimal national and local advertising expenditures in the Stackelberg game is lower than that in the cooperative game. In the two games, both the optimal advertising expenditure and the order quantity are dependent on the advertising efficacy coefficients.

In addition, we show that the properly designed revenue-cost-sharing contract can achieve supply chain coordination and lead to a Pareto improving win-win situation for channel members. Under the revenue-cost-sharing contract, the manufacturer's share of his own advertising expenditure equals the share of the retailer's advertising expenditure and the share of the retailer's revenue he gains. We believe the main challenge for managers is to create acceptance of new types of royalty payments such as a retailer's participation rate in a manufacturer's national advertising expenditure. Both parties should jointly elaborate and implement interorganisational operations and processes that specifically incorporate such payments to eliminate distortions from vertical externalities. The existence of trust in an existing manufacturer-retailer relationship may be helpful to overcome these acceptance problems.

Finally, the Eliashberg's model is used to solve the bargaining problem. Eliashberg's model cannot only consider individual supply chain members' risk preferences but also incorporate supply chain members' negotiating powers into the ultimate implementation outcome. Through the numerical studies, we further confirm that the joint decisions improve the channel performance greatly.

There are several possible directions for the future studies. First, one can involve more decision-makers to enrich the results and add competitive characteristics to the model. Second, one can adopt a different form of demand function. Finally, other bargaining schemes may be applied in order to achieve different results.

## Figures and Tables

**Table 1:** The ranges of parameters.

Parameters	*a*	*b*	*k*_1_	*k*_2_	*c*_*r*_	*c*_*m*_
Ranges	[1000, 10000]	[1.2, 2.5]	[0.4, 2.5]	[0.4, 2.5]	[1, 10]	[10, 50]

**Table 2:** Six groups of values of parameters considered.

Groups	*a*	*b*	*k*_1_	*k*_2_	*c*_*r*_	*c*_*m*_
Group 1	2000	1.5	1.2	1.0	1.0	40
Group 2	4000	1.8	1.0	0.6	5	20
Group 3	5000	2.0	0.8	1.2	4	30
Group 4	3000	1.6	0.6	0.5	8	45
Group 5	8000	2.2	2.0	1.2	6	25
Group 6	6000	1.9	1.0	1.5	3	15

**Table 3:** The equilibrium solutions in the two game settings.

Game structures	Groups	*p*	*q*	*e*	*n*	Π_*r*_	Π_*m*_	Π_*S*_	Δ (%)
Cooperative	Group 1	250.0	80.0	2359.3	1638.4	—	—	3997.7	83.1
	Group 2	87.5	70.8	813.5	292.8	—	—	1106.3	60.8
	Group 3	102.0	14.5	75.9	170.9	—	—	246.8	126.7
	Group 4	229.7	16.0	416.3	289.1	—	—	705.4	81.8
	Group 5	82.7	25.7	244.4	88.0	—	—	332.4	59.8
	Group 6	58.0	443.1	1363.3	3067.5	—	—	4430.8	129.0

Stackelberg	Group 1	520.7	12.8	1132.8	53.2	1541.7	642.1	2183.8	—
	Group 2	145.1	15.6	362.0	13.2	444.8	243.1	687.9	—
	Group 3	168.7	1.9	27.8	9.8	77.2	31.7	108.9	—
	Group 4	438.3	2.7	191.7	10.9	267.7	120.3	388.0	—
	Group 5	119.3	6.2	101.4	4.9	128.3	79.6	207.9	—
	Group 6	99.8	55.1	513.1	164.1	1383.6	551.1	1934.7	—

**Table 4:** The effect of variation of *k*_1_and *k*_2_ on the equilibrium solutions in the two game settings.

Game structures	*k*_1_	*k*_2_	*k*_2_/*k*_1_	*p*	*q*	*e*	*n*	*n*/*e*	Π_*r*_	Π_*m*_	Π_*S*_	Δ (%)
Cooperative	0.8	1.5	1.875	87.5	150.4	520.6	1830.3	3.516	—	—	2350.9	153.7
	1.0	1.0	1.000	87.5	104.1	813.4	813.4	1.000	—	—	1626.9	93.8
	1.2	0.5	0.417	87.5	88.0	1171.4	203.4	0.174	—	—	1374.7	48.1

Stackelberg	0.8	1.5	1.875	163.8	15.1	190.8	93.2	0.488	691.0	235.5	926.5	—
	1.0	1.0	1.000	150.0	17.1	343.4	38.1	0.111	572.3	267.1	839.4	—
	1.2	0.5	0.417	143.6	21.8	530.0	9.0	0.017	587.6	340.4	928.0	—

*a* = 4000, b = 1.8, c_r_ = 5, c_m_ = 20.

**Table 5:** The effect of variation of *b* on the equilibrium solutions in the two game settings.

Game structures	*b*	*p*	*q*	*e*	*n*	Π_*r*_	Π_*m*_	Π_*S*_	Δ (%)
Cooperative	1.4	150.0	1524.6	35031.8	12611.4	—	—	47643.2	62.1
	1.8	87.5	70.8	813.5	292.8	—	—	1106.3	60.8
	2.2	66.7	3.3	25.6	9.2	—	—	34.8	59.8

Stackelberg	1.4	344.8	270.9	17999.1	331.0	20928.0	8466.6	29394.6	—
	1.8	145.1	15.6	362.0	13.2	444.8	243.1	687.9	—
	2.2	96.2	0.8	10.6	0.5	13.4	8.4	21.8	—

*a* = 4000, *k*_1_ = 1.0, *k*_2_ = 0.6, *c*_*r*_ = 5, *c*_*m*_ = 20.

**Table 6:** The allocation results of the extra joint profit.

Bargaining power	ϕ_*r*_	ϕ_*m*_	λ*	ΔΠ_*r*_	ΔΠ_*m*_	ΔΠ
*λ*_1_ = 0.4	0.2	0.6	0.3160	311.9	106.5	418.4
*λ*_2_ = 0.6	0.5	0.5	0.4093	208.8	209.6	418.4
	0.6	0.2	0.5026	105.5	312.9	418.4
*λ*_1_ = 0.5	0.2	0.6	0.3156	312.4	106.0	418.4
*λ*_2_ = 0.5	0.5	0.5	0.4089	209.2	209.2	418.4
	0.6	0.2	0.5022	106.0	312.4	418.4

## Appendices

### A. Proof of Theorem 1

Proof For given *q*, *n*, and *e*, from (5) the optimal stocking factor satisfies the following first order condition:

(A.1) ∂ΠT(q,z,n,e)∂z=(ag(n,e))1/bq1−1/bbz1−1/b ×(1−∫Az[(b−1)xz+1]f(x)dx)=0.

Hence, *z*_*T*_* satisfies

(A.2) $$\begin{array}{c} \overset{z}{\underset{A}{\int }}\left[\frac{\left(b-1\right)x}{z}+1\right]f\left(x\right)dx=1. \end{array}$$

Note that (A.2) is equivalent to (7). When *dh*(*x*)/*dx* > 0, we define

(A.3) G(z)∶=z−∫Az[(b−1)x+z]f(x)dx=z[1−F(z)]−(b−1)∫Azxf(x)dx.

Thus, we have

(A.4) G′(z)=[1−F(z)][1−bh(z)],G′′(z)=−h(z)G′(z)z−b[1−F(z)]h′(z),

where *h*(*z*) ≡ *zf*(*z*)/(1 − *F*(*z*)). From (A.4) and *dh*(*z*)/*dz* > 0, we get *G*′′(*z*) < 0 at *G*′(*z*) = 0, which implies *G*(*z*) itself is a unimodal function. This, in conjunction with

(A.5) $$\begin{array}{c} G\left(A\right)=A\geq 0,\;\;G\left(B\right)=-\left(b-1\right)\overset{B}{\underset{A}{\int }}xf\left(x\right)dx<0, \end{array}$$

guarantees the uniqueness of *z*_*T*_* and *A* < *z*_*T*_* < *B*. From (7), we find that *z*_*T*_* is unrelated to *q*, *n*, and *e*. For given *n* and *e*, it is easy to verify that Π_*T*_(*q*, *z*_*T*_*, *n*, *e*) in (5) is concave with respect to *q*. As a result, the optimal order quantity *q*_*T*_* is uniquely determined by the following first order condition:

(A.6) $$\begin{array}{c} \frac{\partial \prod_{T}\left(q,z_{T}^{*},n,e\right)}{\partial q}={\left[az_{T}^{*}g\left(n,e\right)\right]}^{1/b}q^{-1/b}\left[1-F\left(z_{T}^{*}\right)\right]-c=0. \end{array}$$

According to (A.6), we get

(A.7) $$\begin{array}{c} q\left(n,e\right)=az_{T}^{*}g\left(n,e\right){\left[\frac{1-F\left(z_{T}^{*}\right)}{c}\right]}^{b}. \end{array}$$

Substitute (A.7) into (5), one can derive

(A.8) ΠT(q(n,e),zT∗,n,e) =azT∗g(n,e)c1−b[1−F(zT∗)]bb−1−n−e =azT∗c1−b(k1e+k2A)[1−F(zT∗)]bb−1−n−e.

It is easy to verify that Π_*T*_(*q*(*n*, *e*), *z*_*T*_*, *n*, *e*) in (A.8) is jointly concave with respect to *n* and *e*, respectively. Setting the first-order partial derivatives of Π_*T*_(*q*(*n*, *e*), *z*_*T*_*, *n*, *e*) with respect to *n* and *e* equal to zero, respectively, we obtain

(A.9) $$\begin{array}{c} \frac{\partial \Pi_{T}\left(q\left(n,e\right),z_{T}^{*},n,e\right)}{\partial e}=\frac{ak_{1}z_{T}^{*}{\left[1-F\left(z_{T}^{*}\right)\right]}^{b}}{2\sqrt{e}\left(b-1\right)c^{b-1}}-1=0,\\ \frac{\partial \Pi_{T}\left(q\left(n,e\right),z_{T}^{*},n,e\right)}{\partial n}=\frac{ak_{2}z_{T}^{*}{\left[1-F(z_{T}^{*})\right]}^{b}}{2\sqrt{n}\left(b-1\right)c^{b-1}}-1=0. \end{array}$$

From (A.9), one knows that (9) and (10) hold. Substitute (9) and (10) into (A.7), one finds that (11) holds. Hence, the proof of Theorem 1 is completed.

### B. Proof of Theorem 2

Proof From (7), one can get

(B.1) $$\begin{array}{c} 1-F\left(z_{T}^{*}\right)=\frac{\left(b-1\right)\left[z_{T}^{*}-\Lambda \left(z_{T}^{*}\right)\right]}{bz_{T}^{*}}. \end{array}$$

Substituting (B.1) into (9), (10), and (12) and comparing them with (15), (16), and (17), we have

(B.2) $$\begin{array}{c} e_{T}^{\det}>e_{T}^{*},\;\;n_{T}^{\det}>n_{T}^{*},\;\;p_{T}^{\det}<p_{T}^{*}. \end{array}$$

Since ∂*D*(*p*, *n*, *e*)/∂*e* > 0, ∂*D*(*p*, *n*, *e*)/∂*n* > 0, and ∂*D*(*p*, *n*, *e*)/∂*p* < 0, from (B.2) one can obtain

(B.3) $$\begin{array}{c} D\left(p_{T}^{*},n_{T}^{*},e_{T}^{*}\right)<D\left(p_{T}^{\det},n_{T}^{\det},e_{T}^{\det}\right). \end{array}$$

Hence, the proof of Theorem 2 is completed.

### C. Proof of Theorem 3

Proof Setting the first-order partial derivatives of Π_*m*_(*w*, *n*) in (23) with respect to *n* and *w* equal to zero, respectively, we have

(C.1) $$\begin{array}{c} \frac{\partial \Pi_{m}\left(w,n\right)}{\partial n}=M\left(w-c_{m}\right){\left(w+c_{r}\right)}^{-b}\frac{k_{2}}{2\sqrt{n}}-1=0, \end{array}$$

(C.2) ∂Πm(w,n)∂w=M(w+cr)−b[k1N(w+cr)1−b+k2n] −bM(w−cm)(w+cr)−b−1 ×[k1N(w+cr)1−b+k2n] +k1MN(1−b)(w−cm)(w+cr)−2b=0.

From (C.1), one can derive

(C.3) $$\begin{array}{c} n_{d}^{*}={\left[\frac{k_{2}M\left(w-c_{m}\right){\left(w+c_{r}\right)}^{-b}}{2}\right]}^{2}. \end{array}$$

Substitute (C.3), (24), and (25) into (C.2) and simplifying, we obtain

(C.4) [w+crw−cm]2+[(b−1)k2−2b+1](w+cr)w−cm  −k2b(b−1)=0,

where *k* = *k*_2_/*k*_1_. Let *y* = (*w* + *c*_*r*_)/(*w* − *c*_*m*_); then we get (26). Substituting (24) and (26) into (C.3), one knows that (27) holds. It follows from (C.4) that *y* satisfies the following equation:

(C.5) $$\begin{array}{c} y^{2}+\left[\left(b-1\right)k^{2}-2b+1\right]y-k^{2}b\left(b-1\right)=0. \end{array}$$

Since *y* is a positive variable, from (C.5) we can get the express of *y* given by (31). By substituting (26)-(27) into (19)–(21), one can derive (28)–(30). Hence, the proof of Theorem 3 is completed.

### D. Proof of Lemma 4

Proof From (31), we can write *y* as

(D.1) $$\begin{array}{c} y=\frac{\left[k^{2}\left(1-b\right)+2b-1+\sqrt{H}\right]}{2}, \end{array}$$

where *H* = *k* ^4^(*b* − 1)^2^ + 2*k* ^2^(*b* − 1)+(2*b* − 1)^2^. To show that *y* > *b*, we need only to verify that 2*y* − 2*b* > 0; that is, $k^{2}(1-b)+2b-1+\sqrt{H}-2b=k^{2}(1-b)-1+\sqrt{H}>0$. Due to

(D.2) H−[k2(b−1)+1]2=k4(b−1)2+2k2(b−1)+(2b−1)2 −[k2(b−1)+1]2=4b(b−1)>0,

then *H* > [*k* ^2^(*b* − 1) + 1]^2^. Thus, we have $k^{2}(1-b)-1+\sqrt{H}>0$. Hence, the proof of Lemma 4 is completed.

### E. Proof of Corollary 5

Proof From (28), (9), (30), and (12), one can easily derive that

(E.1) $$\begin{array}{c} e_{d}^{*}={\left[\frac{y-1}{y}\right]}^{2(b-1)}e_{T}^{*}<e_{T}^{*},\;\;p_{d}^{*}=\left[\frac{y}{y-1}\right]p_{T}^{*}>p_{T}^{*}. \end{array}$$

From (27), (10), (29), (11), and Lemma 4, we have

(E.2) nd∗=[y−1y]2b[b−1y−1]2nT∗<nT∗,qd∗=qT∗(y−1)2b−1[yk12+(b−1)k22][y2b(k12+k22)]<qT∗(y−1)2b−1y(k12+k22)[y2b(k12+k22)]=qT∗[y−1y]2b−1<qT∗.

Hence, the proof of Corollary 5 is completed.

### F. Proof of Property 2

Proof By taking the first-order derivative of *y* in (D.1) with respect to *k* results in

(F.1) $$\begin{array}{c} \frac{dy}{dk}=\frac{k\left(b-1\right)}{\sqrt{H}}\left[k^{2}\left(b-1\right)+1-\sqrt{H}\right]. \end{array}$$

It is easy to verify that $k^{2}(b-1)+1-\sqrt{H}<0$. Hence, we have *dy*/*dk* < 0. By the chain rule, from (26) we have

(F.2) $$\begin{array}{c} \frac{dw^{*}}{dk}=\frac{dw^{*}}{dy}\cdot \frac{dy}{dk}=-\frac{c}{{\left(y-1\right)}^{2}}\cdot \frac{dy}{dk}>0. \end{array}$$

From (30), we get

(F.3) $$\begin{array}{c} \frac{dp_{d}^{*}}{dk}=\frac{dp_{d}^{*}}{dy}\cdot \frac{dy}{dk}=-\frac{c}{{\left(y-1\right)}^{2}\left[1-F\left(z_{d}^{*}\right)\right]}\cdot \frac{dy}{dk}>0. \end{array}$$

Hence, the proof of Property 2 is completed.
